# Smartphone Apps to Support Coordinated Specialty Care for Prodromal and Early Course Schizophrenia Disorders: Systematic Review

**DOI:** 10.2196/16393

**Published:** 2019-11-12

**Authors:** Erica Camacho, Leonard Levin, John Torous

**Affiliations:** 1 Beth Israel Deaconess Medical Center Harvard Medical School Boston, MA United States

**Keywords:** smartphones, mobile phones, app, schizophrenia

## Abstract

**Background:**

Demand for mental health services, especially for clinical high-risk and early psychosis, has increased, creating a need for new solutions to increase access to and quality of care. Smartphones and mobile technology are potential tools to support coordinated specialty care for early psychosis, given their potential to augment the six core roles of care: case management and team leadership, recovery-oriented psychotherapy, medication management, support for employment and education, coordination with primary care services, and family education and support. However, the services smartphones are actually offering specifically for coordinated specialty care and the level of evidence are unknown.

**Objective:**

This study aimed to review the published literature on smartphone technology to enhance care for patients with prodromal and early course psychosis and schizophrenia and to analyze studies by type, aligned with coordinated specialty care domains.

**Methods:**

A systematic literature search was conducted on August 16 and 17, 2019, using the PubMed, EMBASE, Web of Sciences, and PsycINFO electronic databases. The eligible studies were reviewed and screened based on inclusion and exclusion criteria.

**Results:**

The search uncovered 388 unique results, of which 32 articles met the initial inclusion criteria; 21 eligible studies on 16 unique app platforms were identified. Feasibility studies showed a high user engagement and interest among patients, monitoring studies demonstrated a correlation between app assessments and clinical outcomes, and intervention studies indicated that these apps have the potential to advance care. Eighteen studies reported on app use for the case management roles of coordinated specialty care. No app studies focused on employment and education, coordination with primary care services, and family education and support.

**Conclusions:**

Although the published literature on smartphone apps for prodromal and first-episode psychosis is small, it is growing exponentially and holds promise to augment both monitoring and interventions. Although the research results and protocols for app studies are not well aligned with all coordinated specialty care roles today, high rates of adoption and feasibility suggest the potential for future efforts. These results will be used to develop coordinated specialty care–specific app evaluation scales and toolkits.

## Introduction

Psychotic disorders are among the most disabling disorders in all of medicine [1]. The costs to society are greater than nearly any other chronic health condition, and the burden to patients and family members is of the greatest magnitude [2-4]. These disorders generally surface during late adolescence or early adulthood [5]. Individuals at clinical high risk are at a greater risk of developing a psychotic disorder; this clinical state is distinguished by impaired functioning, decreased quality of life, and subthreshold psychotic symptoms [6]. Similarly, first-episode psychosis is characterized by a decline in social functioning, the onset of psychotic symptoms like unusual thoughts, hallucinations, and impaired cognitive abilities [7]. Early treatment for clinical high risk and first-episode psychosis is a global health priority that aims to prevent or mitigate the burdens of the illness [8,9]. In this paper, we explore the available evidence for smartphone technology to augment clinical high risk and first-episode psychosis care.

Coordinated specialty care is an evidence-based, recovery-oriented treatment program designed to transform outcomes in first episode psychosis by promoting shared decision making and creating individualized treatment plans. The major roles of care include case management and team leadership, recovery-oriented psychotherapy, medication management, employment and education support, coordination with primary care services, and family education and support [10]. In the United States, there are currently 236 coordinated specialty care programs offering care for first-episode psychosis. Scaling up coordinated specialty care to reach more patients and reduce the duration of untreated psychosis is the next step in expanding services to those with early course psychosis.

One means to augment coordinated specialty care is leveraging technology like smartphones [11]. The rapidly evolving literature on smartphone apps for psychosis spectrum illnesses and a recent review on these apps already suggests its many potential roles in supporting facets of coordinated specialty care [12]. Specific advances around digital phenotyping and just-in-time adaptive interventions hold especially unique promise. By automatically quantifying patients’ treatment trajectories through digital phenotyping, that is, “the moment-by-moment quantification of the individual-level human phenotype in situ using data from personal digital devices” [13], mobile technology could uniquely help offer functional assessments of recovery and personalization of care. These technologies can also potentially help screen and identify those with clinical high risk and ensure appropriate referral of patients with first-episode psychosis to coordinated specialty care that would reduce the duration of untreated psychosis. Through symptom surveys, therapy-based coaching, peer-to-peer support, and medication reminders, smartphone apps have direct potential to support coordinated specialty care today with just-in-time adaptive interventions.

Patients with clinical high risk and first-episode psychosis are already using smartphones and technology today. A 2014 survey conducted on 67 individuals with first-episode psychosis revealed that 88% of the participants had access to phones [14]. Research on smartphone ownership among youth receiving early psychosis–related services suggests that like the rest of the population, people with early psychosis increasingly own devices; a 2015 study reported 81% ownership [3] and 2018 research from our team reported 85% ownership [4]. These studies showcase the feasibility of using technology in care services, as many at clinical high risk and in first-episode psychosis today are already actively using these devices. Given that the clinical needs of patients with early course psychosis are different from those who have had the disease for many years, key roles in coordinated specialty care are customized for early course illness. As these younger patients may use smartphones and technology differently, it is necessary to consider how mobile health may uniquely meet the needs of patients with early course psychosis.

This review paper presents the first of three steps to explore the potential of apps for coordinated specialty care. The next stage will be guided by this review and involves the creation of specialized app evaluation scales, toolkits, and implementation guidelines for coordinated specialty care that will focus on five domains: (1) barriers to application, (2) sustainability following initial implementation, (3) training requirements/burden, (4) necessary infrastructure required to support integration, and (5) sustainable and durability of the technology. The final stage will include eight coordinated specialty care site visits to gather feedback from multiple stakeholders, assess the current state of technology readiness and need, and offer recommendations for successful technology implementation.

There is a clear potential for smartphones and mobile technologies to augment clinical high risk and first-episode psychosis care [12]. In this systematic review, we explore the literature on clinical high risk and first-episode psychosis care, with the goal of assessing feasibility around monitoring and interventions, aligning apps with core roles of coordinated specialty care, and identifying emerging trends. Given the nascent and heterogeneous nature of this space, we respect that different technologies, study designs, populations, and outcomes will preclude formal analysis and permit narrative synthesis.

## Methods

### Overview

A PICO (P - adolescents between the ages of 13 and 26 years, I - mobile/smartphone apps, C - no technological intervention, O - management of prodromal and early course schizophrenia and psychosis) and searchable question was created around the use of mobile technologies in an adolescent population experiencing prodromal and early course schizophrenia and psychosis. Search strategies were developed by author LL, who is a health sciences librarian. LL translated the search strategies based upon each database’s respective controlled vocabulary (Medical Subject Headings, Emtree, Web of Science topic terms, Thesaurus of Psychological Index Terms for PsycINFO) and command language, using additional free-text terms when appropriate. Major concepts searched were *Schizophrenia* and *Psychotic Disorders* combined with *Early Diagnosis* and then joined to a list of applicable terms and synonyms indicating the use of mobile or wireless devices, smartphones, or apps. PubMed, EMBASE, Web of Sciences, and PsycINFO searches were conducted on August 16 and 17, 2019. Criteria included material written in English; age range of 13 through 26 years (adolescent and young adult); and material published from the beginning of 2008, the year in which a medical category was first introduced into the Apple platform app store.

A total of 525 articles were originally discovered. Following the elimination of duplicates, a total of 388 references were identified. Two authors (EC and JT) reviewed each citation/abstract using the Covidence (Melbourne, Australia) systematic review management tool. Inclusion criteria comprised publication date of January 1, 2008, or later; English language; adolescents between the ages of 13 and 26 years exhibiting prodromal, early course, or first-episode schizophrenia or at a high risk for clinical psychosis; and usage or the availability of a mobile/wireless/smartphone app. The following papers were excluded: articles not published in English, review articles, conference papers or poster abstracts, and any technology not delivered via a mobile device such as a desktop computer program or website.

### Study Selection

**Figure 1:**
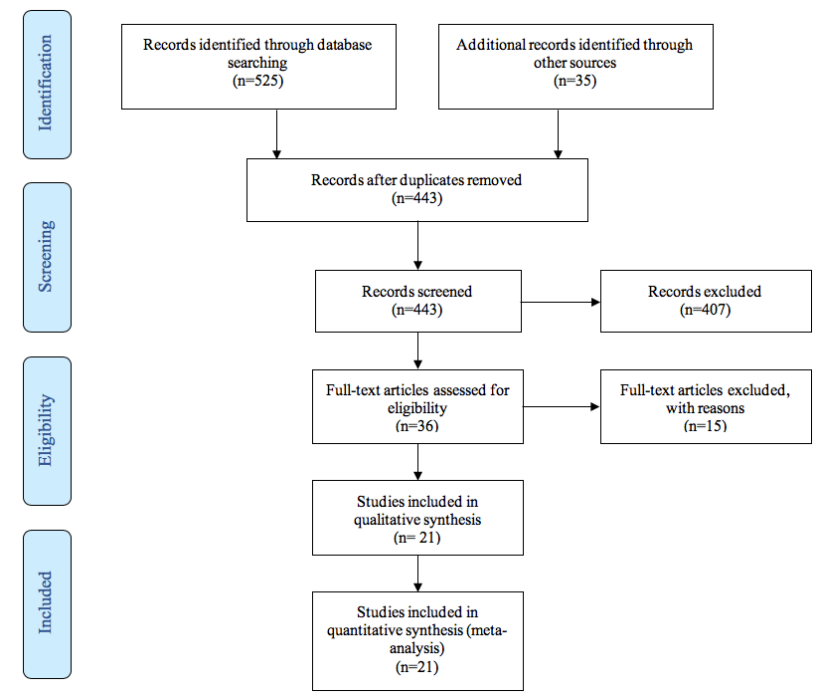
Preferred Reporting Items for Systematic Reviews and Meta-Analyses (PRISMA) flow diagram.

Review of the 388 references was conducted by reviewing each abstract. Each reviewer appraised each article independently. If the abstract was not present or unclear, the full text of the article was retrieved. Following the initial screening, 32 articles met the initial inclusion criteria. Using bibliographies and cited-by references in these papers, hand searching was conducted. In addition, a set of text words culled from these papers was searched in Google Scholar in order to identify any additional grey literature. This process identified 35 additional articles. After full-text review of this final set as well as the original 32 articles, 21 articles met the full inclusion criteria and are reviewed here (Figure 1).

## Results

### Clinical High Risk

#### Protocol

The Robin Z app aims to reduce at-risk symptomatology and comorbid diagnosis while improving functioning, self-efficacy, and quality of life by offering elements of cognitive behavioral therapy, systematic therapy, and self-assessment between therapy sessions. Robin Z features include information and suggestions on coping with their symptoms, medication reminders, crisis intervention planning created with a therapist, weekly goals, and a library of positive reinforcements. Traber-Walker et al [15] plan to evaluate the app in a controlled study with 30 participants aged 14-18 years.

#### Monitoring

The ClinTouch app, one of the first in the psychosis space, collects ecological momentary assessment data from smartphones. The 2012 ClinTouch study lasted for 6 days and split patients into 3 groups: (1) patients in partial or full remission, (2) patients acutely psychotic, and (3) patients at ultra-high risk. Of the 44 participants entered in the study, 6 acute and 2 remitted patients did not complete the minimum number of diary entries and were excluded from analysis. No ultra-high risk participants were excluded. The ultra-high risk group completed more surveys (31.1) during the study than the remitted (29.5) or acute (28.5) group [16]. The mean age of the 12 ultra-high risk participants was 22 years, and 10 of 12 were male. The results suggest that smartphone ecological momentary assessment has high internal consistency and sensitivity to change in all groups, including ultra-high risk.

#### Intervention

MOMENTUM seeks to enrich social functioning in patients at clinical high risk via incorporation of the self-determination theory of motivation and a strength- and mindfulness-based approach. In a 2-month study [17] with 13 clinical high-risk patients (mean age 20.3) and in the 2019 study [18], the program could be accessed on a computer, tablet, or smartphone. The study represents the first efforts to offer interventions for high clinical risk. Results demonstrated a statistically significant improvement in social functioning for all participants, measured on the Global Functioning Scale, and 42% of participants showed improvements in the Satisfaction With Life scale [17].

### First-Episode Psychosis

#### Protocol

Actissist is a digital intervention that focuses on five major domains associated with relapse in early psychosis: (1) perceived criticism, (2) socialization, (3) cannabis use, (4) paranoia, and (5) auditory verbal hallucinations. Smartphone notifications are sent at “3 psuedorandomized time points per day, 6 days a week between the hours 10am to 10pm” to encourage the participant to access the app [19]. This 2015 study explored feasibility and acceptability in patients with first-episode psychosis and demonstrates the app’s ability to reduce psychotic symptoms and cannabis misuse while enhancing their quality of life. Outcomes of this now completed study are illustrated under the intervention section for first-episode psychosis.

The TechCare study [20] will blend experiential sampling methodology, which examines context and flow of “thoughts, feelings, and events,” with intelligent real-time therapy, which delivers psychological interventions such as cognitive behavioral therapy. In this three-phase study, the first phase will involve collecting qualitative data through focus groups, which will be used to refine the intervention for the next phase. This second phase will evaluate the acceptability of TechCare. The third and final phase will be a feasibility trial, where case coordinators will help create personalized interventions by customizing the app and linking ecological momentary assessment responses to treatment options including a crisis plan [20].

HORYZONS is an internet-based platform that was developed to prevent relapse and improve social functioning in users. The platform aims to support long-term recovery in first-episode psychosis by offering therapy modules and features that promote behavior change and social networking. This study protocol adapts earlier foundational work [21] from Australia to now meet the cultural needs of patients with first-episode psychosis in Canada [22]. The program can be accessed via a smartphone, and the study goals are to gain insight on the acceptability of the platform and discover recommendations on how to improve it.

HORYZONS will be utilized for a randomized controlled trial in Melbourne to test whether its use for first-episode psychosis can enhance social functioning and reduce hospitalizations when compared to treatment as usual. Alvarez-Jimenez et al [23] plan to recruit 170 participants with first-episode psychosis, aged 16-27 years, to partake in the 18-month study. The study will utilize ecological momentary assessment to assess positive effect, negative effect, and social isolation via surveys eight times a day, within a 12-hour window. It also features “online pathways” designed to improve participant self-efficacy such as “fostering positive emotions” and “identifying and exercising personal strengths” and is supported by both peer and clinician moderators. Expert moderators, that is, clinicians, will also be able to customize the app and treatment approaches to each patient’s needs.

When planning the Psychotherapy app, Barbeito et al [24] wrote a protocol for a focus group, to gain insight into the opinions of young people with psychosis in Spain [24]. The app, comprised of five modules outlined in Table 1, will be studied to assess its ability to decrease the number of relapses and rehospitalizations in 50 patients with first-episode psychosis, aged 14-19 years, when compared to treatment as usual. Features of the app will be supported by first-episode psychosis users such as the contact wall, while others will be moderated and customized by clinicians.

**Table 1:** Studies of smartphone apps for people with first-episode psychosis or at clinical high risk.

Parameter and intervention/app		Author, year	Main findings
**CHR^a^ - Protocol**			
	Robin Z	Traber-Walker et al, 2019 [15]	The Robin Z app offers support between therapy sessions with the goal of improving the daily functioning of people in CHR states. The study goal is to decrease at-risk symptoms like delusions, depression, and hallucinations following usage of the app in addition to TAU^b^.
**CHR - Monitoring**			
	ClinTouch	Palmier-Claus et al, 2012 [16]	The ClinTouch app offers symptom assessments for CHR patients and provides clinical information to their providers remotely. The study concluded that smartphones apps are a valid method for symptom management as seen from high participant compliance rates.
**CHR - Intervention**			
	MOMENTUM	Alvarez-Jimenez et al, 2018 [17]	The MOMENTUM app is designed to improve the self-efficacy of people at ultra-high risk for psychosis by helping participants focus on their strengths, practice mindfulness, and connect with one another. Results demonstrated improvements in social functioning and wellness as well as high engagement and satisfaction with the app.
**FEP^c^ - Protocol**			
	Actissist	Bucci et al, 2015 [19]	Actissist is an intervention that focuses on five domains that are associated with early psychosis relapse. The study will compare it to ClinTouch, a symptom monitoring app, plus TAU.
	TechCare	Husain et al, 2016 [20]	TechCare blends experiential sampling methodology and intelligent real-time therapy to provide participants with both assessments and interventions. The study collected user feedback to refine the intervention and to test the feasibility of the app.
	HORYZONS	Lal et al, 2018 [22]	HORYZONS is a Web platform, accessible via a smartphone, that is capturing feedback from Canadian youth on the framework with the purpose of adapting the program to better serve those with FEP.
	HORYZONS	Alvarez-Jimenez et al, 2019 [23]	HORYZONS will utilize a smartphone ecological momentary assessment tool to deliver surveys and interactive therapy content to FEP participants with a focus on improving social functioning.
	Psychotherapy	Barbeito et al, 2019 [24]	The Psychotherapy app study will investigate whether five modules in the app may minimize relapse and hospitalization in FEP compared to TAU. The modules include psychoeducation, recognition of symptoms and prevention of relapses, problem solving, mindfulness, and a contact wall.
	MOMENTUM	Vitger et al, 2019 [18]	MOMENTUM is converted into a smartphone app to be utilized for FEP. The emphasis of this study will be on improving shared decision making between patients and their carers.
**FEP - Usability/feasibility**			
	Unnamed app	Smelror et al, 2019 [25]	Smelror et al conducted an exploratory study for the use of an app to assist patients with early onset psychosis to manage their auditory verbal hallucinations.
	PRIME	Schlosser et al, 2016 [26]	PRIME is an intervention app for FEP that provides patients with goal-setting tools, CBT^d^-based coaching from a clinician, and social networking opportunities with their peers. The study showed high engagement rates, 100% retention, and high user satisfaction to conclude that the app is feasible and acceptable.
	Heal Your Mind	Kim et al, 2018 [27]	The Heal Your Mind app offers case management and symptom monitoring for young people with early psychosis. The surveys collected showed that a majority of participants used at least 5 of the 6 modules, felt the app was easy to use, and expressed satisfaction with the tool.
	+Connect	Lim et al, 2019 [28]	+Connect is an intervention app designed to target loneliness in youth with early psychosis. The study outcomes showed a decrease on the University of California Loneliness scale.
	ACT-DL^e^	Vaessen et al, 2019 [29]	The ACT-DL app utilizes acceptance and commitment therapy to help patients with early psychosis improve negative symptoms. The study showed that participants found the app to be a useful tool to solidify knowledge gained from weekly therapy sessions.
	RealLife Exp	Kuman et al, 2018 [30]	The RealLife Exp app is used alongside a Web-based dashboard to help early psychosis patients with symptom monitoring. Study outcomes indicate that participants are moderately responsive to daily and weekly assessments.
**FEP - Monitoring**			
	CrossCheck	Ben-Zeev et al, 2017 [31]	The CrossCheck app collects ecological momentary assessments, device use data, and passive data like geolocation to predict relapse in people with psychosis. Study outcomes indicate that digital indicators of relapse are not the same for every individual experiencing psychosis.
	mindLAMP	Wisniewski et al,2019 [32]	The mindLAMP app also collects ecological momentary assessments, device use data, and passive data like geolocation. Study outcomes indicate that digital markers can help inform changes in clinical care.
	Ginger.io	Niendam et al, 2018 [33]	Ginger.io is a symptom-monitoring app for individuals with early psychosis, which collects survey responses and passive data like distance travelled and phone calls. The study showed that the app is easy to use and a willingness of patients to continue incorporating apps into the patient’s treatment plan.
	ClinTouch	Cella et al, 2019 [34]	ClinTouch is used alongside a wearable device to draw conclusions on whether there is a connection between distressing psychosis symptoms and physiological responses. The study outcomes showcase increased electrodermal activity when experiencing hallucinations or delusions, but no association between symptoms and heart rate variability.
**FEP - Intervention**			
	PRIME	Schlosser et al, 2018 [35]	The PRIME app seeks to improve motivation in FEP. The study findings show improvements in reward learning, anticipated pleasure, and effort expenditure for the PRIME group as compared to the waitlist.
	Actissist	Bucci et al, 2018 [36]	The Actissist app plus TAU showed greater and more sustained treatment effects and benefits as compared to a symptom monitoring app plus TAU. A majority of participants from the Actissist arm submitted at least half of their data entries, and all members of this arm were retained.

^a^CHR: clinical high risk.

^b^TAU: treatment as usual.

^c^FEP: first-episode psychosis.

^d^CBT: cognitive behavioral therapy.

^e^ACT-DL: acceptance and commitment therapy in daily life.

The 2019 MOMENTUM protocol aims to assess how app use in patients with first-episode psychosis can augment shared decision making with their providers. With a planned enrollment at 260 participants aged 18-35 years, it represents the largest sample size planned to date [18]. The app will be used by the participant to explore and evaluate aspects of their daily life such as sleep and stress and then share those data with the providers. By increasing the patients’ knowledge of and comfort with their symptoms in parallel with the providers’ increased awareness of daily changes, the study hypothesizes that app use will facilitate shared decision making in first-episode psychosis.

#### Usability/Feasibility

Smelror et al [25] conducted an exploratory study for 7 days by using an app to assist patients with early onset psychosis to cope with their auditory verbal hallucinations. Of the four patients contacted to participate in the study, one refused and one was excluded from the statistical analyses due to poor compliance. Of note, one participant reported discomfort with digital monitoring and noted feeling watched, monitored, and insecure. The study concluded that adolescents with early onset psychosis are willing to use apps to self-assess their symptoms and apps are a viable option for this population.

The PRIME app is an intervention tool, supported by peers and coaches, which aims to improve quality of life and negative symptoms like motivation in first-episode psychosis. In this two-phase study, the first 10 enrolled participants (mean age 23.4) used the app to determine feasibility. Based on phase one feedback, the second phase was an ongoing randomized controlled trial with the first 10 participants (mean age 23.3) testing the effectiveness of the app’s iterative design process. Over 12 weeks, participants logged in about every other day and completed 1.5 daily challenges a week, such as working out for 30 minutes. Retention was high at 100%, and the average level of satisfaction for all participants was 8/10 [26]. The study concluded that PRIME is a feasible and highly acceptable intervention tool for first-episode psychosis.

The Heal Your Mind app provides cognitive behavioral therapy–based case management and symptom monitoring for young people with first-episode psychosis through six modules: (1) thought record, (2) symptom record, (3) daily life record, (4) official notices, (5) communication, and (6) scales [27]. This feasibility study involved 33 early intervention service users (mean age 25.6). Usage reports were high, with 41.7% of participants completing all 6 modules [27]. The most frequently used, liked, and perceived helpful feature was communication with the case manager. High satisfaction was reported in about 80% of participants and 46% felt the app was useful for monitoring their symptoms. One participant reported feelings of stress while using the app. The study concluded that the app is feasible and useful for young people experiencing early psychosis.

The +Connect app delivers positive psychology interventions via three different video types: peer videos, expert videos, and actor videos. Videos are offered in response to real-time mood tracking. The 6-week study required 12 participants (mean age 20.5) to complete the daily activities on the app for at least 70% of the duration. Both user engagement and satisfaction were high at 95.47% and 90%, respectively [28]. Conclusions of the study were that the app is highly acceptable, feasible, and usable.

The acceptance and commitment therapy in daily life (ACT-DL) study [29] utilized a subset of participants to determine the feasibility of an ongoing randomized controlled trial, which plans to enroll 150 participants into the program. The ACT-DL app offers ACT toward improving negative symptoms. In this clinical hybrid study, participants meet with ACT therapists weekly and then apply the lessons learned on the app platform for a minimum of 3 consecutive days. Participants are prompted to answer a short questionnaire on their mood and symptoms. The app then elicits an ACT metaphor or exercise based on the module of the week. The 16 participants to date found both the ACT therapy sessions and the home exercises useful. They also reported that the app helped them implement ACT into their daily life and helped bring awareness to their emotions [29].

The RealLife Exp app provides symptom monitoring for patients with early psychosis through daily and weekly smartphone surveys. The 5-month study involved 61 participants with early psychosis interacting with the app and 20 providers who connected through the online dashboard. Of the 41 participants who completed the study, 66% reported a willingness to continue using RealLife Exp as part of their treatment service, while 12% reported a lack of interest in using the app [30]. A majority of participants suggested improvements to the app, such as technical functionality, product enhancements, and changes to the survey.

#### Monitoring

A case series paper [31] of the CrossCheck app featured two patients with first-episode psychosis, aged 19 years, likely meeting. The CrossCheck app combines ecological momentary assessment with digital phenotyping (smartphone-based) geolocation, speech frequency and duration, and physical activity. The app was preinstalled on smartphones with an unlimited data plan provided to participants in the study. With a 12-month data-collection period, it represents the longest reported use of a mobile health smartphone app for people with early psychosis. In the first case, a 19-year-old African-American male with schizophrenia had ecological momentary assessment data for 91% of the 125 days before hospitalization and audio sensor data for 87% of the days. The audio sensor data showed a decrease in speech frequency and duration over 50 days and then an increase over 70 days, followed by a spike in both before hospitalization. In the second case, a 19-year-old Hispanic American-Indian female with schizophrenia had ecological momentary assessment data for 91% of the 190 days prior to hospitalization and device use data for 93% of the days. Device use data showed that the patient’s phone was unlocked between midnight and 6 AM during the 60 days before hospitalization, which was unusual compared to her first 100 days of data [31]. Similarly, our team wrote a case series exploring the role of passive data monitoring in patients with psychosis, including first-episode psychosis, using the mindLAMP app [32].

Ginger.io offers another platform for both ecological momentary assessment and digital phenotyping. In this clinical hybrid study, including 64 participants with recent-onset psychosis and 12 with clinical high risk, the app collected active data through self-report surveys and digital phenotyping including phone calls, messages, and distance traveled [33]. As a clinical hybrid, the study involved monthly in-person psychosocial assessments with the research team. A total of 97% percent of the 60 participants who completed satisfaction surveys found the app easy to use, and 83% were open to continued use of the app in their treatment plan [33]. The authors report that smartphone assessments of symptoms were comparable to the Brief Psychosis Rating Scale conducted at the monthly clinician interviews.

The 2019 ClinTouch study [34] was a 10-day observational cohort study exploring the association between symptoms of psychosis, such as hallucinations and delusions, to physiological responses, such as heart rate variability and electrodermal activity. The study used a wearable, E4, to measure physiological changes and used the mobile app to conduct the self-assessment. Smartphone notifications were sent at “4 pseudo-randomized time points per day between the hours 11am to 9pm” to prompt participants to rate their symptoms [34]. One participant dropped out due to a non–research-related reason. Of the 14 participants who completed the study, 76% of surveys were completed [34]. As distressing hallucinations and delusions were reported, there was a significant increase in electrodermal activity. No significant association was found between these symptoms and heart rate variability [34]. Participants found the app to be easy to use, nondisruptive, and enjoyable overall.

#### Intervention

The 2018 PRIME study [35] conducted a 12-week randomized controlled trial to test the app’s ability to enhance motivational impairments in first-episode psychosis. Of the 43 participants recruited (mean age: 24.3), 22 were in the PRIME group, 21 were in the treatment as usual/waitlist group, 5 dropped out, and 6 did not complete the follow-up. To assess changes in motivated behavior, the trial modified the Trust Task to determine three aspects of motivation: reward learning, anticipated pleasure, and effort expenditure. Only PRIME participants were given the Trust Task. These participants showed a greater increase in anticipated pleasure, effort expended to increase the likelihood of future social interactions with positive outcomes, and learning from positive outcomes from baseline to 12 weeks compared to waitlist [35]. The retention rate for the treatment was 74%, and mean satisfaction with PRIME was 8.21/10. Participants noted that the app helped them feel less alone, more hopeful, more connected, and less helpless. The ability to directly message their coaches was the most popular feature, while the ability to track mood was the least popular.

The 2018 Actissist proof-of-concept study [36] involved a 12-week randomized controlled trial for 36 participants. The study had a 2:1 ratio for the experimental group Actissist plus treatment as usual (n=24, mean age 20 years) to the control group ClinTouch plus treatment as usual (n=12, mean age 18 years). The study set a target criterion of half of the participants in each arm to submit at least half of the data entries. The Actissist arm met the target criterion (63%), while ClinTouch did not (42%) [36]. In addition, 75% of Actissist users engaged with the app at least once a day. The most popular prompted and unprompted domain was voices followed by suspicious thoughts. The Actissist arm showed larger treatment effects and greater benefits than symptom monitoring plus treatment as usual in the immediate posttreatment assessment and the 22-week follow-up.

## Discussion

### Principal Findings

In this systematic review, we examined the published literature on smartphone apps for both prodromal and first episode psychosis and found 21 studies. Seven papers were protocols, six were feasibility studies, five were monitoring/validity studies, and three were interventions. The heterogeneous outcomes of the 21 studies and 16 unique app platforms utilized preclude formal analysis, and data on engagement, adherence, and feasibility varied greatly, as outlined in the narrative results. Overall, feasibility studies were positive and reported high engagement and interest among patients, monitoring studies showed that apps captured outcomes correlated to and informative of clinical outcomes, and intervention studies demonstrated that these apps have the potential to advance care.

Every study aligned with at least one core role of coordinated specialty care but none aligned with all. Of the 21 studies, 18 incorporated case manage mentor team leadership, 12 involved recovery-oriented psychotherapy, and 7 mentioned medication management. A majority of these apps offer case management through crisis planning and symptom monitoring. This finding aligns with recent reviews [12]. Recovery-oriented psychotherapy in these apps was offered by both clinicians and peers. None of the studies reported provision of support for employment and education, coordination with primary care services, or family education and support, as seen in Textbox 1. This does not mean that apps cannot support coordinated specialty care; instead of a single app offering resources to meet all needs, a toolkit approach of several apps may be more feasible today. Although many apps for adult psychosis exist and could offer services for individual roles of coordinated specialty care, it is unclear if such apps designed and assessed in adult patients would equally benefit youth.

Many of the apps reviewed offered social and peer support as their main feature or intervention [17,22-24,26,35]. For example, HORYZONS incorporates peer moderators in the “café” feature where youth with mental illness can share their lived experiences to help participants navigate issues they may be facing. PRIME users expressed positive feedback from the social support they received from peers and their coaches. Other apps provide space for peer interaction, such as MOMENTUM and the Psychotherapy app. The Psychotherapy app uniquely provides two avenues for peer contact, including both more formal peer support and less formal peer interactions. One implementation challenge in moving promising research toward peer-to-peer forums is the need for moderation and safety mentoring, which can become difficult at scale.

Clinicians involved with these smartphone apps can have a variety of roles, such as moderating social interactions, reviewing patient clinical status, and customizing patient interventions. For example, through the Psychotherapy app and HORYZONS, clinicians can review user data to create personalized content suggestions. Ginger.io alerted clinicians when surveys were not completed for more than 3 days in a row, when passive data were not being collected, or when participant responses were considered clinically significant. The MOMENTUM study hypothesizes that an increased awareness of daily changes in the patient will better equip both parties for shared decision making [18]. These apps utilize their programs as a clinical hybrid to better fit the patient’s needs. Implementation of such a hybrid model of care will involve helping clinicians optimally work with technology, a focus of future efforts of this ongoing initiative.

The potential of smartphone apps for care is, in part, driven by their scalability, and our study results support the potential for sharing and reusing apps. The HORYZONS trial that adapted the Australian app to meet the treatment needs of youth in Canada, offers an example of global accessibility and collaboration possible through digital tools. The ClinTouch app has been used not only for feasibility studies of symptom reporting and physiological markers, but also for a control app in intervention studies, highlighting the multiple uses of these apps. It may be possible to use apps not directly targeting clinical high risk and first-episode psychosis; such a toolkit will also be the next focus of this initiative.

**Coordinated specialty care checklist.**

Case management and team leadership: n=18

Recovery-oriented psychotherapy: n=13

Medication management: n=7

Supported employment and education: n=0

Coordination with primary care services: n=0

Family education and support: n=0

Although many studies focused on innovations in monitoring patients or interventions, few offered both. Results from numerous studies suggest that it is possible to capture self-reported symptoms via apps that are comparable to clinical assessments, and the 2019 ClinTouch study demonstrates how this can even be linked to physiology. Although the CrossCheck studies demonstrated the potential utility of app monitoring via digital phenotyping to predict relapse, no studies instigated the predictive validity of app monitoring. The TechCare study offers an example of a combined approach as well as how clinicians and smartphone apps can work symbiotically. The intervention portion of the app is tailored to the participant’s survey responses and the specific delusions they are experiencing. If the patient is in a low mood or paranoid state for a prolonged period, the agreed-upon crisis plan is deployed. Tailoring interventions based on digital phenotyping data today would be more speculative, given the current lack of strong data, suggesting a reliable clinical interpretation of those data.

The growing interest in smartphone apps for prodromal and first-episode schizophrenia is reflected in the high number of recently published protocol papers we identified. These newly planned studies intend to expand the size and duration of completed ones, with a mean of 29.8 participants in completed studies compared to a targeted mean of 84.1 participants from protocols, as seen in Table 2. Future studies aim to increase the study duration by almost double to a mean duration of 5.7 months as compared to completed studies with a mean duration of 2.9 months. Not all planned, or even currently underway, studies published protocols; therefore, these results must be interpreted with some caution, although they offer insight into designing and powering the next wave of relevant studies.

The results of this review also suggest the low risk of harm in using app tools in patients with prodromal and first-episode schizophrenia. No adverse events were reported in any study, although some participants reported higher stress levels due to the fear of being watched or monitored [25]. This is likely not unique to their conditions, as across the general population, there is a small percent who are also not comfortable using technology and feel concerned about digital monitoring [37]. Some apps reported on security measures in place to protect privacy such as the Robin Z app, which requires fingerprint scanning or a pin code to login, but this was not reported consistently across studies.

Like all studies, this review has several limitations. First, no search term can identify all relevant papers on this topic and many publications are hard to recognize, given the varied nosology around prodromal, first-episode, and smartphone tools. Second, we used clinical judgment in including some studies such as the case report on CrossCheck, as not every paper directly identifies its population as first episode. Third, more apps and studies may exist that have not published protocols or data papers, meaning that our results are influenced by publication bias. We aimed to minimize these limitations by working with a librarian to build the search term and conduct the search.

**Table 2:** Summary metrics for studies on smartphone apps for people with first-episode psychosis or at clinical high risk.

Metric	Value	Studies
Studies in the United States, n (%)	6 (29)	21 studies [15-20,22-36]
Participants enrolled in completed studies, mean	29.8	12 studies [16,17,25-30,33-36]
Participants planned to be enrolled, mean	84.1	12 studies [16,17,25-30,33-36]
Participants dropped out from completed studies, mean	3.4	12 studies [16,17,25-30,33-36]
Age in completed studies (years), mean	21.5	11 studies [16,17,25-28,30,33-36]
Completed studies with male as the majority gender, n (%)	7 (70)	10 studies [16,17,26-28,30,33-36]
Duration of completed studies (months), mean	2.9	12 studies [16,17,25-30,33-36]
Duration of planned studies (months), mean	5.7	7 studies [15,19,20,22-18]

### Conclusions

Results of this systematic review suggest that while the published research on smartphone apps for prodromal and first-episode psychosis is nascent, it is rapidly expanding and holds the potential to improve both monitoring and interventions. Although we did not find a single app that aims to fulfill all key roles of coordinated specialty care, these results are useful for understanding the current impact of mobile technology for care and future trends. The next steps in our work supporting such technology for coordinated specialty care will involve creating an evaluation and implementation framework that will help programs select and utilize a toolkit composed of several apps. By assessing these frameworks at coordinated specialty care sites across the country, we will learn which digital tools and implementations may best help sites augment care for patients with early course psychosis.

## Appendix

Multimedia Appendix 1 Inclusion and exclusion criteria for Table 2.

## Abbreviations

ACT-DL: acceptance and commitment therapy in daily life
CBT: cognitive behavioral therapy
CHR: clinical high risk
FEP: first-episode psychosis
PICO: P - problem/patient/population, I - intervention/indicator, C - comparison, O - outcome
TAU: treatment as usual
