# Supplementary material for: Smartphone Apps to Support Coordinated Specialty Care for Prodromal and Early Course Schizophrenia Disorders: Systematic Review
Source: J Med Internet Res. 2019 Nov 12;21(11):e16393. doi: 10.2196/16393 (PMC6880233; doi:10.2196/16393)
Supplement: Multimedia Appendix 1 [file jmir_v21i11e16393_app1.pdf]

Appendix 1

The 21 studies discussed in this review are compared quantitatively in Table 1. The ‘mean number enrolled in completed studies’ category excludes case series in addition to protocols seeing as case reports are only indicative of a subset of participants involved in the study. The same is true for other categories in the table including 1) mean dropped out from completed studies, 2) mean age in completed studies, 3) completed studies with male as the majority gender, and 4) average duration of completed studies. The ACT-DL study did not include the demographics of the 16 participants [29], therefore, this study was excluded from both the ‘mean age in completed studies’ and ‘completed studies with male as the majority gender’ categories. Smelror et al. did not disclose the gender of their participants; for this reason, the study was excluded from the ‘completed studies with male as the majority gender’ section [25].
